# Spatial Distribution and Determinant Factors of Handwashing Practice With Essential Agents Among Households in Ethiopia

**DOI:** 10.3389/ijph.2022.1604040

**Published:** 2022-04-12

**Authors:** Sewnet Adem Kebede, Biruk Shalmeno Tusa, Adisu Birhanu Weldesenbet

**Affiliations:** ^1^ College of Medicine and Health Sciences, University of Gondar, Gondar, Ethiopia; ^2^ College of Health and Medical Sciences, Haramaya University, Dire Dawa, Ethiopia

**Keywords:** Ethiopia, household, handwashing practice, essential agents, spatial distribution

## Abstract

**Objectives:** This study aimed to assess the spatial distribution and determinant factors of handwashing practice using essential handwashing agents (soap and water) among households in Ethiopia.

**Methods:** A two-stage stratified cluster sampling technique was used. Mixed-effect logistic regression analysis was also used to identify determinants of handwashing practice with essential agents.

**Results:** In Ethiopia, household handwashing practices with essential agents had spatial variation (Moran’s Index 0.62, *p* < 0.001). The Amhara and Somali regions were identified as significant hotspots with low handwashing practice using essential agents.

**Conclusion:** In Ethiopia, handwashing practice with essential agents showed spatial variation across the country with a very low rate. Areas with low handwashing practice with essential agents need high priority in the allocation of resources to ensure communities’ access to fixed and portable handwashing facilities, soap, and reliable water supplies. Households with low access to improved sanitation facilities, low wealth status, and low educational status should be targeted for the intervention.

## Introduction

Handwashing is defined as the brief, vigorous rubbing together of all surfaces of lathered hands followed by rinsing under a stream of water. It is the act of cleansing hands using soap and water or using an antiseptic hand rub for the removal of transient microorganisms from the hands. Hand hygiene is a simple, easily implemented, and effective practice, and is a significant contributor to the prevention and control of infectious diseases, particularly water- and foodborne and feco–orally transmitted diseases [1–3].

Handwashing with soap and water (handwashing with essential agents) is one of the most effective measures against infectious diseases. Handwashing removes pathogenic agents and reduces the number of microbes on the hands, which could minimize the spread of germs and thereby prevent diarrhea, acute respiratory infections like influenza, and skin infections [4, 5]. Handwashing reduces the spread of infectious diseases, which reduces the frequency of childhood death. Reductions in pneumonia, diarrhea, and measles collectively were responsible for half of the 3.6 million fewer deaths recorded in 2013 worldwide [6].

Globally, 2.3 billion people lack basic sanitation [7]. About 842,000 deaths are attributed to inadequate Water Sanitation and Hygiene (WASH) each year, representing 58% of total diarrheal deaths [8]. The problem of poor hygiene is worse in developing countries where WASH is one of the most important needs within public health. In sub-Saharan Africa, WASH remains one of the major public health challenges with very low coverage [9]. About 58% of the population in sub-Saharan Africa does not use WASH facilities and lacks basic drinking water, and only 15% has access to handwashing facilities that include soap and water [7]. In Ethiopia, studies showed only 15% of schoolchildren reported handwashing after defecation [10].

Reducing the number of child deaths in those aged 5 years and below is one focus of Sustainable Development Goals (SDGs) [11] To this end, improving hand hygiene is of paramount importance because contaminated hands increase the transfer of pathogens, which may cause illness via hand contact. Handwashing practice is considered to be influenced by several factors. Most notably, educational status, the ethnicity of the household head, accessibility and availability of water and soap, household wealth, access to an improved sanitation facility, residence, and access to an improved water source [12–14] are factors associated with handwashing practice.

Inadequate and inefficient infrastructures for water, sanitation, housing, and personal hygiene facilities are responsible for such an unacceptable burden of diseases and deaths [15]. Existing research and government reports of Ethiopia showed that 29% of the Ethiopian population has access to basic water, while only 7% has access to basic sanitation [16]. Ethiopia, Nigeria, and the Democratic Republic of Congo account for one-third of the people in Sub-Saharan Africa who do not have access to basic handwashing facilities at home [17]. Despite the overwhelming evidence in favor of handwashing with soap and water in many low- and middle-income countries, many people do not have soap and water to wash their hands or clean with, and the practice of handwashing at key moments is not widespread. Nearly three out of every four people in the least-developed countries have nowhere at home to wash their hands with soap and water. This creates a higher risk of spreading illness and disease, including coronavirus. To curb the spread of COVID-19 in these countries, this must change [18].

The Government of Ethiopia, with support from several development partners and Non-Governmental Organizations (NGOs), launched the One WASH National Program in 2013 [19]. The government of Ethiopia also promotes global handwashing day (15 October), which was declared by UNICEF in 2008, and they use this to highlight the importance of washing your hands with soap as an effective and affordable way to prevent diseases [20]. Sustainable Development Goal 6 includes a target to ensure everyone has access to adequate and equitable hygiene by 2030. Progress towards that target will be measured by the proportion of the population using safely managed sanitation services, including a handwashing facility with soap and water [21, 22].

Furthermore, hand hygiene practices, including important handwashing agents, vary by region [23–26], and to the author’s knowledge, no national study has looked into the spatial distribution and determinant factors of hand hygiene practices using essential handwashing agents in Ethiopia. Thus, this study aimed to assess handwashing practices using essential handwashing agents among households in Ethiopia. The finding of this study is helpful for both policymakers and healthcare providers to implement targeted strategies to minimize infections caused by poor hygiene, such as COVID-19, by prioritizing areas that had a low occurrence of handwashing practices with essential agents.

## Methods

### Study Design and Area

The Ethiopian Demography and Health Survey (EDHS) 2016 was used to conduct secondary data analysis. Every 5 years, the EDHS is conducted in Ethiopia’s nine regional states [Afar, Tigray, Amhara, Benishangul-Gumuz, Gambella, Harari, Oromia, Somali and Southern Nations, Nationalities, and People’s Region (SNNPR)] as well as two administrative cities (Addis Ababa and Dire-Dawa) [27]. Ethiopia’s current population, according to Worldometer elaboration of the most recent United Nations data, was 115,114,480 as of Friday, 24 July 2020 [28].

### Data Source and Study Period

Data for this study, including geographic coordinates (longitude and latitude coordinates), were obtained from the official database of the Demography Health Survey (DHS) program, www.measuredhs.com, after permission was granted *via* an online application that explained the study’s aim. The EDHS 2016 household data (HR) set was used. Between 18 January and 27 June 2016, Ethiopia conducted the 2016 Ethiopian Demographic and Health Survey (EDHS) [27].

### Sampling Procedure, Study Population, and Sample Size

Using the 2007 Population and Housing Census as a sampling frame, the EDHS 2016 used a stratified two-stage cluster sampling technique. In the first stage, 645 enumeration areas (EAs) were carefully selected with a probability proportional to the EA size and an independent selection in each sampling stratum (202 in urban areas and 443 in rural areas). In the second stage, 28 households were systematically chosen. The full EDHS 2016 report included a detailed sampling technique [27]. The total weighted samples of 9,966 households were included in this study.

### Study Variables

The dependent variable for this study was the “presence of handwashing practice with essential agents in the households.” In 2016 EDHS, to obtain handwashing information, interviewers asked to see the place where members of the household most often wash their hands. Soap and water, the essential handwashing agents, were observed in households. The dependent variable was then divided into two groups: “Yes” if a household had experienced handwashing with essential agents and “No” if a household did not experience handwashing practices with essential agents within the study period. The explanatory variables included in the study were residence, the number of household members, the sex of the household head, improved water source, educational status of household head, improved sanitation facility, region, wealth quintile, and place where household members wash their hands.

### Operational Definitions

Handwashing practice with essential agents: this was defined as the brief, vigorous rubbing together of all surfaces of lathered hands with soap and water. Soap and water are essential handwashing agents [29].

Improved sanitation facilities: this was defined as one that hygienically separates human excreta from human contact. This includes flush or pour-flush toilets flowing to a piped sewer system, septic tank, or latrine, ventilated pit latrine, pit latrine with slab, and composting toilet. The type of sanitation facility used by each household was classified as having or not having an improved sanitation facility [30, 31].

Improved water sources: this was defined as whether the household used piped water (into dwelling, compound, yard, or plot, piped to a neighbor, public tap/standpipe), tube well/borehole, protected well, protected spring and rainwater collection for drinking purposes. If bottled water was used, the households must have had to use any of the improved water sources listed above for other purposes, such as cooking and washing hands, to be considered as using improved water sources [31, 32].

### Data Processing and Analysis

Before any statistical analysis, the data were weighted to restore the generalizability of the survey and take into account the sampling design, and get reliable statistical estimates. We used STATA 14 software to show descriptive statistics and summary statistics in the form of text and tables.

### Spatial Analysis

Exploring the spatial distribution, global spatial autocorrelation, and identifying major hotspot areas of handwashing practices with essential agents were carried out using ArcGIS version 10.3 and SaTScan version 9.6 statistical software.

### Spatial Autocorrelation and Spatial Interpolation

The presence of spatial autocorrelation was determined using the global Moran’s index (Moran’s I). Moran’s I values range from −1 to 1 [33]. A value near 1 denotes a significant positive spatial autocorrelation, whereas a value near −1 denotes a strong negative spatial autocorrelation. There is no spatial autocorrelation if Moran’s I is close to 0. A statistically significant Moran’s I value (*p <* 0.05) can lead to rejection of the null hypothesis (handwashing practice with essential agents is randomly distributed) and show the presence of spatial autocorrelation.

### Spatial Scan Statistical Analysis

Using Kuldorff’s SaTScan software, a Bernoulli-based model was fitted to find statistically significant spatial clusters of handwashing practice with essential agents. Households with experience of handwashing with essential agents was taken as cases and those with no experience of handwashing practice with essential agents were considered as controls to fit the Bernoulli model. The default maximum spatial cluster size of *<*50% of the population was used.

For each potential cluster, a Likelihood Ratio (LR) test statistic and the p-value were applied to identify if the number of observed cases within the potential cluster was significantly higher than expected or not. The scanning window with maximum likelihood was the most probable performing cluster, and the p-value was assigned to each cluster using Monte Carlo hypothesis testing by comparing the rank of the maximum likelihood from the real data with the maximum likelihood from the random data sets. Based on 999 Monte Carlo replications, the most performing cluster and the secondary clusters were ranked by using their likelihood ratio test [34].

### Generalized Linear Mixed Model

According to EDHS data, households in one cluster may be more similar to one other than households in another cluster. This contradicts the assumptions of observational independence and equal variance across clusters. This implies the use of advanced models to account for between-cluster heterogeneity. Due to the dichotomous nature of the response variable, binary logistic regression and a Generalized Linear Mixed Model were fitted. Deviance Information Criteria were used to compare the models (DIC). A mixed-effect model with the lowest DIC was chosen. The Intra-class Correlation Coefficient (ICC) value was 0.2, which told us to select the Generalized Linear Mixed Model over the binary logistic regression model.

After adjusting the effect of other variables, variables with p-values ≤0.2 in the bi-variable analysis were fitted in the multivariable analysis to quantify the effect of each variable. Adjusted Odds Ratio (AOR) with a 95% Confidence Interval (CI) and p-value < 0.05 in the multivariable model was declared as determinant factors of handwashing practice with essential agents. A variance inflation factor (VIF) was also used to assess multi-collinearity.

## Results

### Sociodemographic Characteristics

This study used a weighted sample of 9,966 households. The percentage of households living in rural areas was 72.55%. The household wealth of 29.14% of the household was in the two poor wealth quintiles, 17.98% were in the middle and 52.86% were in the two upper wealth quintiles. The majority of the households (70.25%) were using an improved water source, but only 19.46% had an improved sanitation facility (Table 1).

**TABLE 1:** The percentage of households with handwashing practice with essential agents in Ethiopia, 2016.

Variables	Weighted Frequency	Percent
Sex of household head		
Male	7306	73.31
Female	2660	26.69
Residence		
Urban	2736	27.45
Rural	7230	72.55
Region		
Tigray	607	6.09
Afar	50	0.50
Amhara	3349	33.60
Oromia	3099	31.10
Somali	191	1.92
Benishangul	113	1.14
SNNPR	1777	17.83
Gambela	31	0.31
Harari	15	0.15
Addis Ababa	688	6.91
Dire Dawa	46	0.46
Improved water source		
Yes	7000	70.25
No	2966	29.75
Improved sanitation facilities		
Yes	1939	19.46
No	8027	80.54
Place where household members wash their hands		
Fixed place	603	6.05
Mobile place	9363	93.95
Household wealth quintile		
Poorest	1268	12.73
Poorer	1636	16.41
Middle	1792	17.98
Richer	1986	19.93
Richest	3284	32.93
Educational level		
No education/preschool	5022	50.39
Primary	2998	30.09
Secondary	978	9.81
Higher	968	9.71

### Spatial Distribution of Handwashing Practice With Essential Agents

**FIGURE 1:**
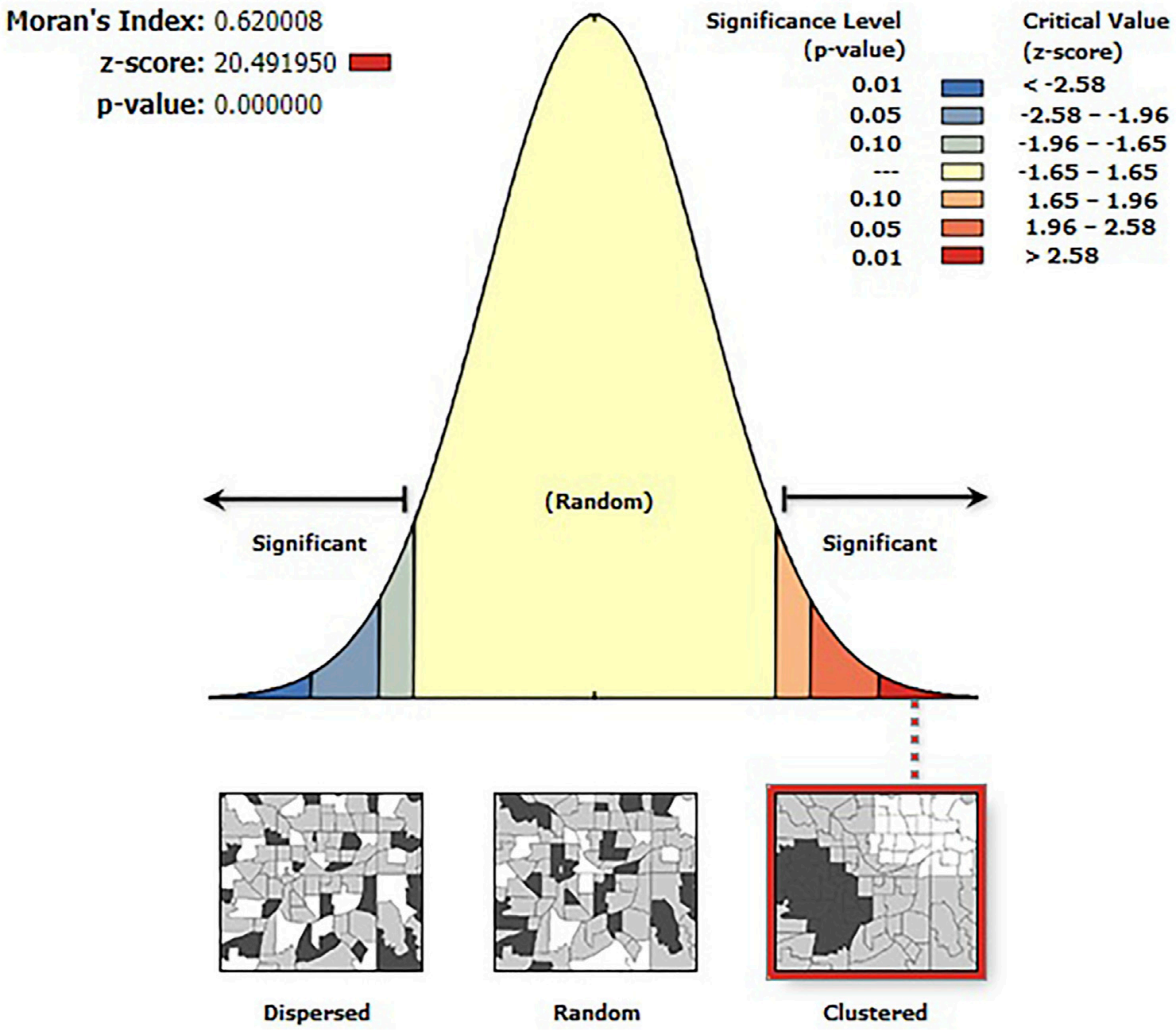
Spatial autocorrelation analysis of handwashing practice with essential agents in Ethiopia, 2016.

According to this study, the spatial distribution of handwashing practice with essential agents was found to be spatially clustered in Ethiopia with a Global Moran’s I 0.62 (*p* < 0.001). A cluster of high rates of handwashing practice with essential agents was observed over the study area. The outputs were automatically generated keys on the right and left sides of each panel. A z-score of 20.49 indicated that there is less than a 1% likelihood that this clustered pattern could be the result of chance. The end tails are bright red and blue, indicating a higher level of significance. (Figure 1).

**FIGURE 2:**
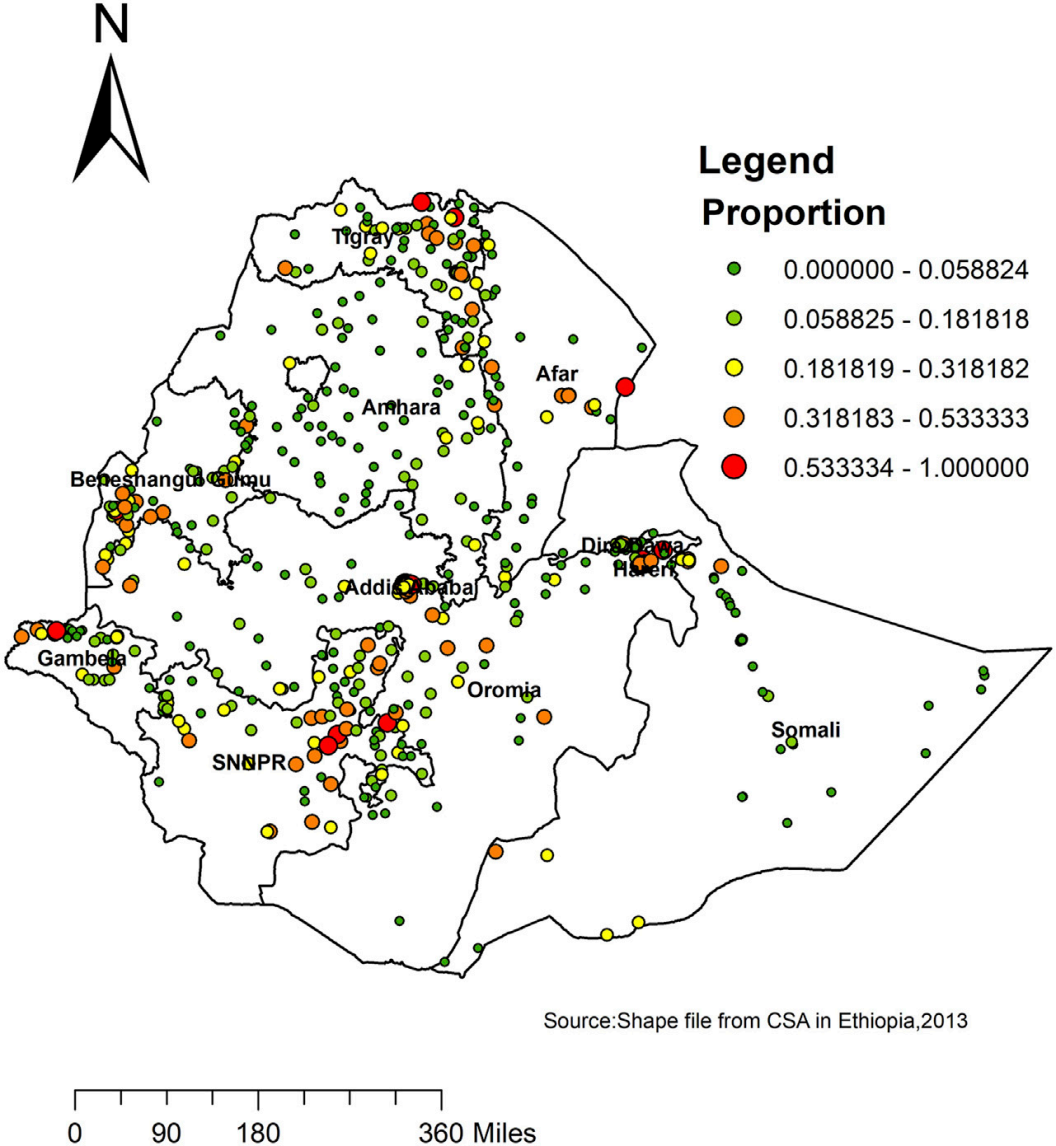
Spatial distribution of handwashing practice with essential agents across regions in Ethiopia, 2016.

Spatial clustering of handwashing practice with essential agents was found at regional levels. Of a total of 9,966 households, only 1,299 (13.03%) households practice handwashing with essential agents. The highest prevalence of household handwashing practice with essential agents was identified in Addis Ababa, Tigray, Afar, Dire Dawa, SNNPR, and Gambella, while the Amhara and Somali regions had the lowest prevalence of household handwashing practice with essential agents (Figure 2).

### Spatial SaTScan Analysis of Handwashing Practice With Essential Agents (Bernoulli Based Model)

Most likely (primary clusters) and secondary clusters of handwashing practice with essential agents were identified. In EDHS 2016, spatial scan statistics identified a total of 61 high and modest performing spatial clusters of handwashing practice with essential agents. Of these, 54 clusters were high performing clusters (LLR = 249.32, RR = 3.08, *p* < 0.001), and 7 clusters were modest performing clusters (LLR = 24.77, RR = 2.68, *p* < 0.001). Addis Ababa, SNNPR, Tigray, Beneshangul, and Afar regions had the highest performing clusters of handwashing practice with essential agents (Table 2).

**TABLE 2:** Significant spatial clusters of areas with a high proportion of household handwashing practice with essential agents in Ethiopia, 2016.

Cluster	Enumeration area (cluster) identified	Coordinate (radius)	Population	Case	RR	LLR	P value
1	274, 11, 463, 339, 91, 532, 369, 107, 626, 31, 100, 108, 144, 112, 635, 305, 170, 153, 414, 582, 195, 314, 59, 464, 15, 487, 645,159,247, 639, 608, 145, 293, 302, 110, 19, 225, 264, 155, 428, 61, 509,560, 451, 539, 330, 287, 211, 261, 475, 402, 90, 147	(9.065960 N, 38.707046 E)/ 19.80 km	1304	539	3.08	249.32	<0.001
2	180, 20, 141, 53, 434, 162, 126	(6.720108 N, 37.624880 E)/ 46.85 km	113	52	2.68	24.77	<0.001

RR: relative risk; LLR: log likelihood ratio.

**FIGURE 3:**
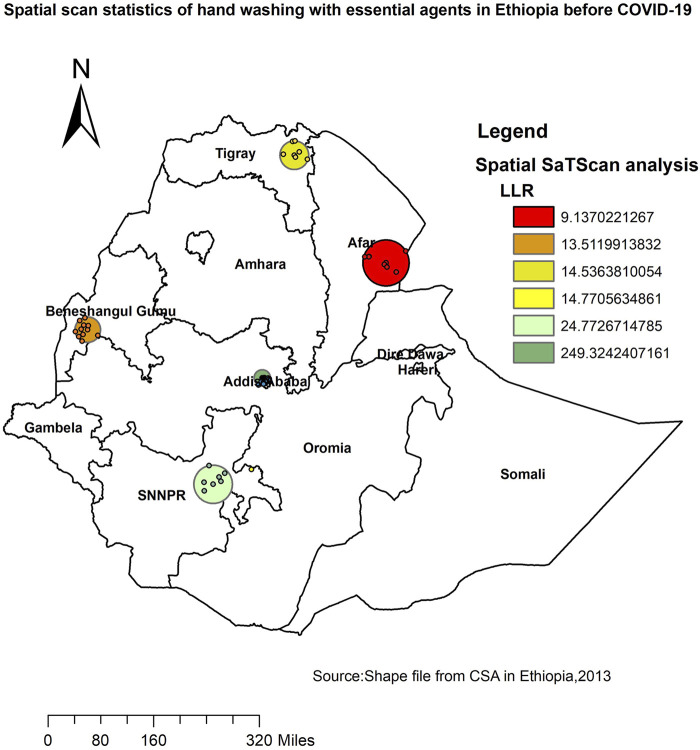
SaTScan analysis of primary and secondary clusters of household handwashing practice with essential agents across regions in Ethiopia, 2016.

The circles represent the most statistically significant spatial windows of handwashing practice with essential agents. There was high handwashing practice with essential agents within the clusters than outside the cluster (Figure 3).

### Factors Associated With Handwashing Practice With Essential Agents in Ethiopia

In the mixed-effect logistic regression model, residence, improved water source, educational status, improved sanitation facility, region, wealth quintile, and place where household members wash their hands were associated with handwashing practice with essential agents in the bi-variable analysis at p-value 0.2. However, in the multivariable mixed-effect logistic regression analysis, educational status, improved sanitation facility, region, wealth quintile, and place where household members wash their hands were significantly associated predictors of households with handwashing practice with essential agents.

The odds of handwashing with essential agents gradually increased depending on the educational status of household heads. The higher the educational status, the more likely it was that household members washed their hands. Accordingly, if the household head had secondary educational status, households were 1.66 times more likely to wash their hands (AOR = 1.66, 95% CI: 1.34, 2.06) compared to households where household heads had no education.

Households where household heads had higher educational status were two times more likely to wash their hands (AOR = 2.00, 95% CI: 1.60, 2.49) compared to households where household heads had no education.

Households with richer wealth quintiles were 1.94 times more likely (AOR = 1.94, 95% CI: 1.35, 2.79) to wash their hands compared with households in the lowest wealth quintiles. Households in the richest wealth quintiles were 2.45 times more likely to wash their hands with essential agents (AOR = 2.45, 95% CI: 1.63, 3.67) compared with households in the lowest wealth quintiles. Households that do not use improved sanitation facilities were less likely to wash their hands with essential agents (AOR = 0.56, 95% CI: 0.46, 0.67).

The odds of washing hands with essential agents were decreased by 81% among households with mobile places where household members wash their hands (AOR = 0.19, 95% CI: 0.16, 0.23) compared with households with fixed places where household members wash their hands. Compared to households in the Tigray region, households in Addis Ababa (AOR = 1.23, 95% CI: 1.08, 1.94) were more likely to wash their hands with essential agents. Compared to households in the Tigray region, households in Amhara (AOR = 0.30, 95% CI: 0.18, 0.48) and Somali (AOR = 0.42, 95% CI: 0.23, 0.76) were less likely to wash their hands with essential agents (Table 3).

**TABLE 3:** Bi-variable and multivariable mixed-effect logistic regression analysis of household handwashing practice with essential agents in Ethiopia, 2016.

Variables	Handwashing with essential agents		Odds Ratio (95% CI)	
	No	Yes	COR	AOR
Residence				
Urban	1976	760	1	1
Rural	6691	539	0.19 (0.15, 0.25)	0.72 (0.51, 1.04)
Region				
Tigray	502	105	1	1
Afar	42	8	0.69 (0.37, 1.31)	0.92 (0.51, 1.66)
Amhara	3177	171	0.19 (0.12, 0.34)	0.30 (0.18, 0.48)
Oromia	2732	367	0.57 (0.34, 0.95)	0.66 (0.42, 1.05)
Somali	178	13	0.36 (0.19, 0.68)	0.42 (0.23, 0.76)
Benishangul	93	20	1.01 (0.59, 1.72)	1.16 (0.72, 1.87)
SNNPR	1449	328	1.19 (0.73, 1.93)	1.54 (0.99, 2.39)
Gambela	24	7	0.73 (0.42, 1.28)	0.67 (0.40, 1.12)
Harari	9	6	1.64 (0.91, 2.93)	0.63 (0.36, 1.09)
Addis Ababa	420	268	4.83 (2.98, 7.82)	1.23 (1.08, 1.94)
Dire Dawa	40	6	0.85 (0.48, 1.50)	0.34 (0.19, 0.58)
Family size				
1–5	5863	955	1	1
6–10	2715	335	0.96 (0.82, 1.12)	0.97 (0.83, 1.14)
≥ 11	89	9	1.42 (0.78, 2.60)	1.18 (0.62, 2.26)
Improved water source				
Yes	1976	760	1	1
No	6691	539	0.65 (0.52, 0.81)	0.94 (0.75, 1.18)
Improved sanitation facility				
Yes	1317	622	1	1
No	7350	677	0.32 (0.27, 0.38)	0.56 (0.46, 0.67)
Educational status				
No education/preschool	4648	374	1	1
Primary	2636	363	1.45 (1.23, 1.72)	1.18 (0.99, 1.42)
Secondary	748	230	2.45 (2.00, 2.99)	1.66 (1.34, 2.06)
Higher	635	332	3.48 (2.85, 4.27)	2.00 (1.60, 2.49)
Wealth quintile				
Poorest	1228	41	1	1
Poorer	1532	104	1.57 (1.08, 2.29)	1.34 (0.92,1.96)
Middle	1676	116	1.79 (1.24, 2.58)	1.36 (0.94, 1.99)
Richer	1796	190	2.85 (2.01, 4.06)	1.94 (1.35, 2.79)
Richest	2435	848	7.25 (5.24, 10.04)	2.45 (1.63, 3.67)
Sex of household head				
Male	6384	922	1	1
Female	2283	377	0.88 (0.76, 1.01)	0.97 (0.84, 1.13)
Place where household members wash their hands				
Observed, fixed place	351	252	1	1
Observed, mobile place	8316	1047	0.15 (0.12, 0.18)	0.19 (0.16, 0.23)

## Discussion

ArcGIS- and SaTScan-based spatial statistical techniques provide an opportunity to clarify and identify household handwashing practice with essential agents within a country and upcoming investigations into predictors responsible for increased handwashing practice with essential agents. Geographic understanding of handwashing practice with essential agents in Ethiopia using these tools was important to target interventions in high-risk areas.

In the current study, the distribution of handwashing practice with essential agents varied from country to country. A Global Moran’s value of 0.62 (*p* < 0.001) indicated that there was a significant clustering of handwashing practice with essential agents in the study area. Our findings showed that only 13.03% of the households practiced handwashing with essential agents in Ethiopia.

The highest prevalence of handwashing with essential agents was reported in the Addis Ababa, SNNPR, Tigray, Beneshangul, and Afar regions, whereas the lowest prevalence of handwashing with essential agents was reported in the Amhara and Somali regions. The multivariable model also revealed consistent findings showing Addis Ababa was the place where the highest handwashing practice with essential agents is recorded, and Amhara and Somali were found to be the place where the smallest number of households practice handwashing with essential agents. A possible explanation for this could be that a minority received formal education (14%) in the Somali region. In the Somali region, the use of improved water sources is low, and the average water transportation time (roundtrip) is significant [35]. The other possible explanation could be that most of the Somali population are mobile pastoralists living far away from villages and water sources. As much as 80% of the total population of the Amhara region lived in rural areas where sanitation-related indicators were low [36].

Another factor that affects handwashing practice with essential agents was the wealth quintile; the odds of handwashing with essential agents were higher among households in the richer and richest wealth quintiles. Similar studies were done in Vietnam [13] and Indonesia [37]. A possible explanation could be that as economic status increases the availability of soap and water also increase. Additionally, wealthier households were more likely to have soap and water in the house, showing that the availability of soap and water increases the rate of handwashing. This finding emphasized the role of socioeconomic factors as potential determinants of handwashing with essential agents. A total of 3 billion people around the world do not have access to clean water and soap, and so this small action to prevent infection remains out of reach for them [38].

Households not using improved sanitation facilities were less likely to wash their hands. This finding is consistent with those from other settings elsewhere [13]. This could be due to healthcare workers from various backgrounds providing health information that covers not only improved sanitation facilities but also the importance of handwashing practice with essential agents.

The educational status of the household head was found to be a predictor of handwashing with essential agents in this study. Household heads with secondary and higher educational levels were more likely to wash their hands with essential agents as compared to household heads with no education. Similar studies have also shown a direct relationship between household head educational status and handwashing practice with essential agents [13, 39, 40]. This could be explained by the fact that educated household heads are more likely to be aware of handwashing practices with essential agents than those without. Furthermore, households head with higher levels of education were more likely to have employment opportunities, which could contribute to a better socioeconomic status in terms of water and soap accessibility and affordability.

The current studies showed that handwashing with essential agents was decreased by 81% among households with mobile places where household members wash their hands as compared with households with fixed places where household members wash their hands. The possible explanation could be households with fixed places where household members wash their hands would be more conscious about handwashing practice with essential agents. The study’s representativeness at the national and regional levels was one of its strengths. Furthermore, statistical analysis using GIS and SaTScan helped to detect statistically high-risk clusters of handwashing practice with essential agents. However, the study did not show the particular locations for data confidentiality reasons. Because the EDHS data were secondary, some potentially important predictors, such as social, behavioral, and cultural factors of handwashing with essential agents, were not included.

### Conclusion

Handwashing practice with essential agents was found to be spatially clustered; the Addis Ababa, SNNPR, Tigray, Beneshangul, and Afar regions had the highest practicing rates, and the Amhara and Somali regions had the lowest practicing rates in this study. Educational status, improved sanitation facility, region, wealth quintile, and place where household members wash their hands were significantly associated predictors of households with handwashing practice with essential agents. Therefore, areas with a low rate of handwashing with essential agents are of high priority for the allocation of resources to ensure communities’ access to fixed and portable handwashing facilities, soap, and reliable water supplies. Households with reduced access to improved sanitation facilities, low wealth status, and low educational status should be targeted for the intervention.

## Abbreviations

AIC, akaike information criteria; AOR, adjusted odds ratio; BIC, Bayesian information criteria; CI, confidence interval; COR, crude odds ratio; CSA, Central statistics agency; DIC, deviance information criteria; EA, enumeration area; EDHS, Ethiopian demographic and health survey; SDG, sustainable development goal; SNNPR, Southern Nation and Nationality and Peoples Region; WASH, water soap and hygiene.
